# Characterization of canine anti-mouse antibodies highlights that multiple strategies are needed to combat immunoassay interference

**DOI:** 10.1038/s41598-019-51228-3

**Published:** 2019-10-10

**Authors:** Daniel Bergman, Anders Larsson, Helene Hansson-Hamlin, Emma Åhlén, Bodil Ström Holst

**Affiliations:** 1 Department of Clinical Sciences, Swedish University of Agricultural Sciences, Uppsala, 750 07 Sweden; 2 Department of Medical Sciences, Uppsala University, Uppsala, 751 85 Sweden

**Keywords:** Laboratory techniques and procedures, Immunology, ELISA, Assay systems

## Abstract

Immunoassays are widely used for detection and quantification of analytes in biological samples, but are vulnerable to analytical errors caused by interfering sample substances. Of particular interest are endogenous anti-animal antibodies that may bind to the immunoassay antibodies and cause erroneous test results. This phenomenon is a hazard to patient safety in both human and veterinary medicine. Here, we demonstrate that anti-mouse antibodies in dogs bind selectively to different regions of the murine IgG molecule, cross-react with IgG from different species, and consist of all major antibody classes present in canine serum (IgA, IgG and IgM). The antibody characteristics varied among individuals and their prevalence differed between two dog breeds. The selective binding to different IgG regions suggests that the antibodies might not originate from immunization through exposure to mice or other species. These findings show that canine anti-mouse antibodies are highly heterogeneous in nature and therefore require a combination of strategies to be counteracted.

## Introduction

The demands for biochemical tests with high analytical sensitivity are increasing within clinical practice and research, as many biologically important analytes are present at low concentrations. At the same time, high analytical specificity is of importance for avoiding false positive results. The immunometric assay is one of few methods capable of satisfying both these needs. It makes use of the interaction between an antibody and its antigen to detect and quantify specific analytes. However, the method is also haunted by fundamental problems that may not be accepted in the presence of cost-efficient alternatives. One such problem is interference from endogenous antibodies in blood, commonly known as anti-mouse antibodies or heterophilic antibodies, which bind to the immunoassay antibodies in presence or absence of the analyte. This is a well-known source of erroneous test results. Although antibodies reactive with mouse antibodies are most frequently described, reactivity to antibodies raised in other species has also been recognized^1–4^. The first report of antibody interference was published in the early 1970s and concerned false positive test results due to endogenous anti-guinea-pig antibodies in human blood donors^5^. Almost 50 years later, new interference reports are still being submitted from all over the world^6–9^.

Despite the long-standing awareness of anti-mouse antibodies, their characteristics are not well-defined. In light of the growing evidence for clinically relevant antibody interference affecting companion animals^10–12^, there is a need for deeper knowledge of the nature of these interfering antibodies in veterinary medicine. This information can be useful for immunoassay manufacturers and clinical laboratories aiming to prevent and manage immunoassay interference. Preventive strategies employed by immunoassay manufacturers target all samples, but strategies employed by laboratories are probably only feasible if they target defined risk groups. If the problem is sufficiently common in a certain patient cohort, such as dogs of a specific breed, proactive countermeasures might even be indicated. There is preliminary data pointing to a potential breed variation in the prevalence of canine anti-mouse antibodies^13^. However, any action taken to reduce the effects of antibody interference would depend on the antibody characteristics, including IgG fragment-specific affinities, cross-reactivity to IgG from a variety of species and immunoglobulin isotype of the anti-mouse antibodies. We therefore analyzed serum samples from two dog breeds, the Bernese mountain dog and the Labrador retriever, in a number of antibody characterizing immunoassays. The Bernese mountain dog is a tentative high-risk breed for interference and the Labrador retriever group represents a comparatively healthy control breed without any apparent risk factors for interference. The goal was to investigate whether there is a difference in the prevalence of anti-mouse antibodies between these two breeds, and to characterize the detected anti-mouse antibodies.

## Results

### Breed difference in prevalence of canine anti-mouse antibodies

The screening for breed differences comprised 110 samples from 104 dogs, 51 Bernese mountain dogs (2 with 2 samples) and 53 Labrador retrievers (4 with 2 samples). The Bernese mountain dog cohort consisted of 25 intact females, 8 neutered females, 11 males and 7 neutered males. Fifteen Bernese mountain dogs (29%) had clinical signs of disease or were diagnosed with disease at the time of sampling. The median age was 3 years [IQR 1.5–6 years]. The Labrador retriever cohort consisted of 14 intact females, 8 neutered females, 28 males and 3 neutered males. Twenty-nine of the Labrador retrievers (55%) were diagnosed with disease or had clinical signs of disease. The median age was 4 years [IQR 2–8 years]. There were significant differences in health status (p < 0.01) and sex (p = 0.02) between the breeds.

Fourteen samples from 12 of the 104 dogs (12%) tested positive for anti-mouse antibodies as determined with the screening ELISA. Ten of 51 Bernese mountain dogs (20%) and 2 of 53 Labrador retrievers (4%) were positive. Two dogs, one Bernese mountain dog and one Labrador retriever, submitted two positive samples. The difference in prevalence between the two breeds was significant (p = 0.03). The median age of positive dogs was 3.5 years [IQR 1.5–7.5 years]. There were 3 each of females, males, neutered females and neutered males. Six of the dogs had some diagnosis or clinical signs of disease at the time of sampling. Individual age, sex and health data for positive dogs is detailed (Table 1).

### Characterization of immunoglobulin binding preferences and isotype properties of anti-mouse antibodies

**Figure 1:**
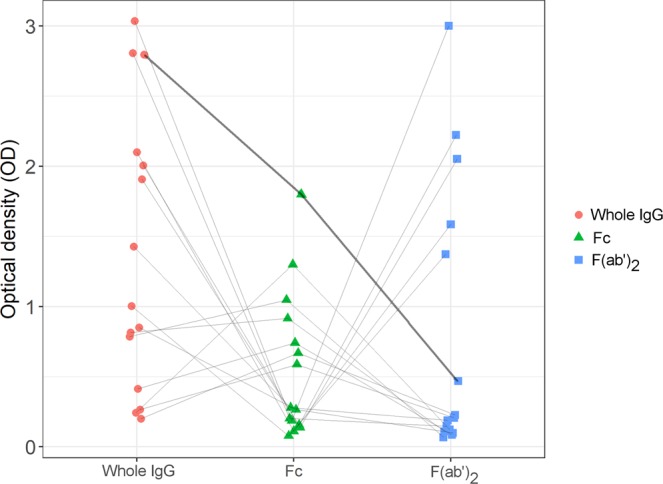
Pattern of reactivity to Whole IgG, Fc- and F(ab’)_2_-fragments raised in mouse. Individual samples are connected by the grey lines. The reference sample (chicken anti-mouse IgG) is indicated by the thickened line.

Out of the 14 positive samples from 12 dogs, five samples from five dogs (42% of the dogs) reacted with whole IgG and F(ab’)_2_-fragments, three samples from three dogs (25%) only with whole IgG, three samples from two dogs (17%) with whole IgG and Fc-fragments and three samples from two dogs (17%) only with Fc-fragments. None of the samples reacted with both Fc- and F(ab’)_2_-fragments. However, the reference sample reacted with both fragments of the IgG molecule and with whole IgG (Fig. 1). The S/N ratio for the whole IgG assay was 11.9, for the Fc assay 9.7 and for the F(ab’)_2_ assay 22.4.

**Table 1:** Summary of the antibody characterization performed in this study.

Breed	Age (years)	Sex	Clinical signs/diagnosis	Whole IgG (mouse)	Fc (mouse)	F(ab’)_2_ (mouse)	Cross-reactivity	Isotype
BMD	3	MN	−	+	−	+	−	IgA, IgG, IgM
BMD	3	FN	−	+	−	−	−	IgG
BMD	1	F	−	+	−	+	−	IgM
BMD	1	F	−	+	−	+	Goat	N/A
LR	8	M	−	+	−	+	−*	N/A
BMD	4	M	−	+	−	−	−	IgM
BMD	5	FN	Lipoma	+	−	+	−	IgA, IgG, IgM
BMD***	8	MN	Protein losing enteropathy	+	+	−	−	IgG
BMD	10	FN	Anterior cruciate ligament injury	+	−	−	Goat, sheep	N/A
BMD	1	F	Gastric dilatation volvulus	+	+	−	−	−
BMD	6	M	Mast cell tumour	−	+	−	−	IgM
LR***	3	MN	Polyuria/polydipsia	−	+	−	−**	N/A

Isotyping was not performed on samples that contained inadequate volumes, or that reacted with goat IgG. Abbreviations: BMD, Bernese mountain dog; LR, Labrador retriever; M, male; F, female; N, neutered. *Not tested for reactivity to chicken IgY. ^**^Not tested for reactivity to rabbit IgG or chicken IgY. ***Two samples from this dog were analyzed.

**Figure 2:**
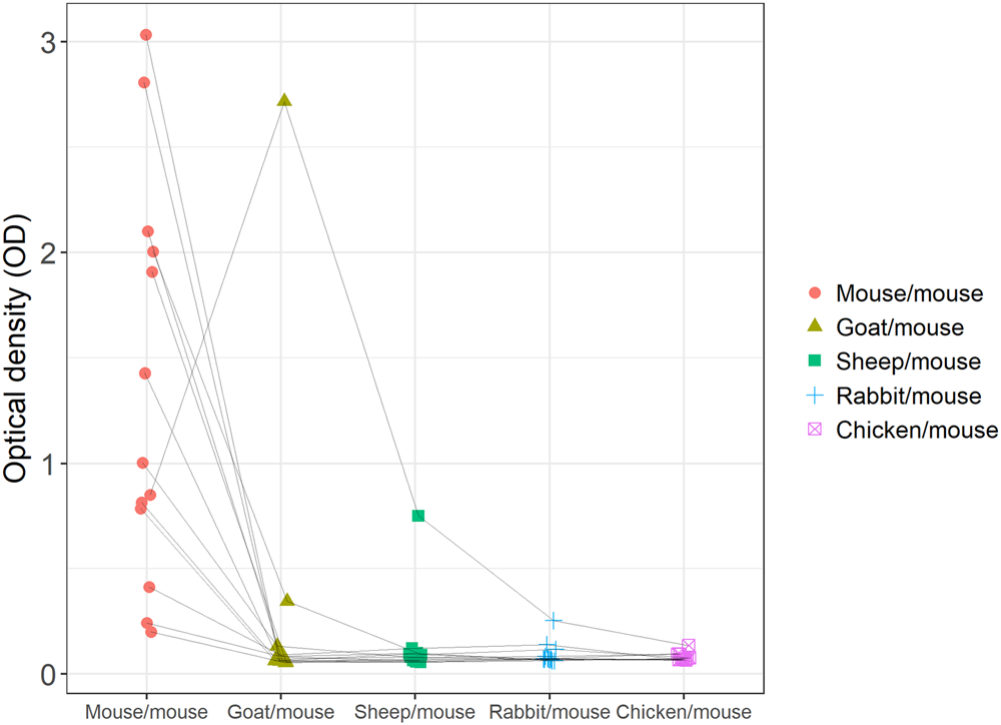
Cross-reactivity of canine anti-mouse antibodies to immunoglobulins of other species. The solid phase of a microtiter plate was coated with 2 µg/mL mouse, goat, sheep and rabbit IgG and chicken IgY, respectively. HRP-conjugated monoclonal mouse IgG (1:200) was used for detection. Individual samples are connected by the grey lines.

The endogenous antibody isotypes and their reactivity to different species of IgG were determined with ELISA. Due to insufficient sample volumes, one sample was not tested in the cross-reactivity experiment with rabbit IgG nor chicken IgY, and one sample was not tested with chicken IgY. Overall, two of the 14 positive samples from 12 dogs (17%) reacted with IgG from multiple species. Two samples from two dogs (17%) cross-reacted with goat IgG and one sample from (8%) with sheep IgG. One sample (8%) cross-reacted with both goat IgG and sheep IgG (Fig. 2). One of the samples cross-reacting with goat IgG was blocked with three blocking solutions; 0.5 mg/mL goat IgG, 0.5 mg/mL mouse IgG, and a mixture of 0.25 mg/mL goat and 0.25 mg/mL mouse IgG. The signal decreased by 96.8% when the sample was treated with goat IgG, by 96.1% with mouse IgG, and by 94.2% with the mixture of goat and mouse IgG.

**Figure 3:**
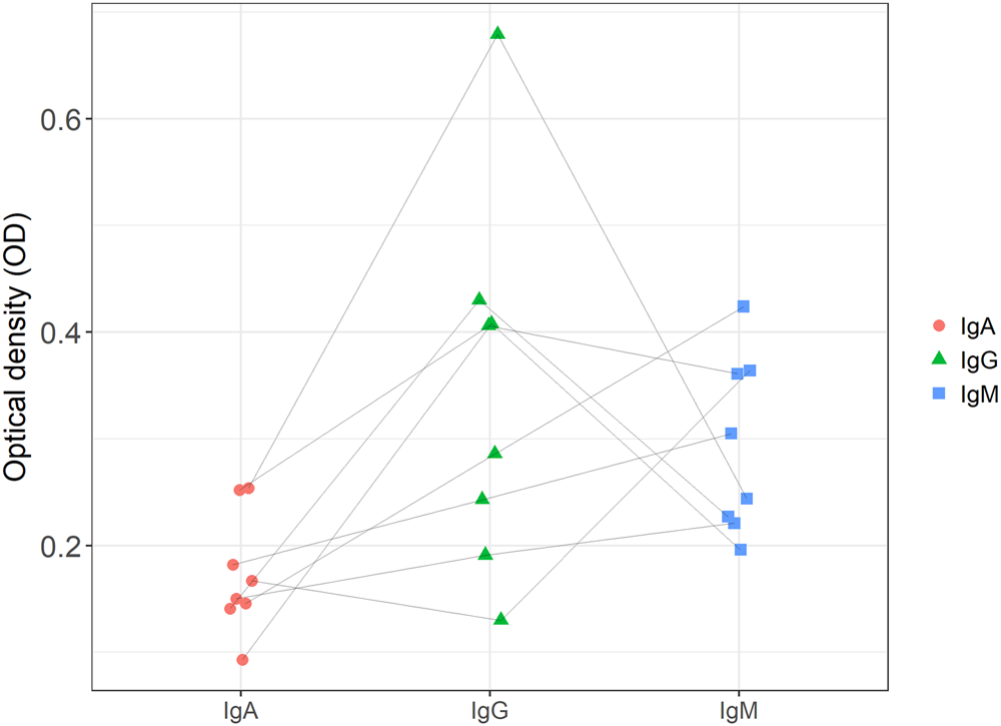
Isotyping of canine anti-mouse antibodies. The wells were coated with 2 µg/mL normal mouse IgG. Endogenous anti-mouse antibodies were detected with 1:10,000 dilutions of Fc-specific biotinylated anti-canine IgA, IgG and IgM, respectively. Individual samples are connected by the grey lines.

Two samples were excluded from the isotyping experiment due to their cross-reactivity to goat IgG, which left eight samples from eight dogs with sufficient sample volumes remaining. Two of the isotyped samples (25%) were positive for IgG only, three (37%) for IgM only, and two (25%) for IgA, IgG and IgM. One sample (12%) was negative for all isotypes (Fig. 3).

## Discussion

The goal of this study was to determine if there is a difference in the prevalence of anti-mouse antibodies between Bernese mountain dogs and Labrador retrievers, and to characterize anti-mouse antibodies in canine serum. This information can be used by immunoassay manufacturers and clinical laboratories to develop strategies for preventing and managing immunoassay interference.

Anti-mouse antibodies were significantly more common in Bernese mountain dogs than in Labrador retrievers. We have previously observed a seemingly disproportionate number of sera with anti-mouse antibodies from Bernese mountain dogs at our laboratory, which might suggest an overrepresentation for Bernese mountain dogs rather than an underrepresentation for Labrador retrievers, compared to the general dog population. However, it is not known whether this predisposition translates to a higher incidence of immunoassay interference in Bernese mountain dogs. The impact of the breed on samples submitted for routine analysis in direct patient care may not be appreciable, unless the immunoassay used to analyze the sample is insufficiently protected against interference from endogenous antibodies. This might be the case with in-house assays set up by researchers and laboratories with custom reagents, as general ELISA guidelines sometimes recommend incubating samples in a simple “dilution buffer”, rather than a blocking buffer containing IgG to overcome interference from endogenous antibodies. Blocking buffers are commercially available, and easily self-made by adding non-immune IgG to any regular diluent of choice. Commercial immunoassay kits are normally equipped with a blocking buffer containing non-immune IgG. This reduces the risk of antibody interference but does not completely solve the problem, as is evidenced by numerous publications^14–16^.

It is not clear why a certain dog breed would be prone to develop anti-mouse antibodies. In veterinary medicine, Bernese mountain dogs are well-known for their high mortality due to neoplastic disease^17–19^, and it has occasionally been shown that human patients diagnosed with cancer are more prone to develop anti-mouse antibodies than patients not diagnosed with cancer^20^. Endogenous antibodies to IgG are more commonly associated with autoimmunity, due to the presence of rheumatoid factors (autoantibodies to the Fc portion of IgG) in patients with autoimmune diseases. However, systemic autoimmune disease in dogs is fairly rare, and Bernese mountain dogs are typically not included in the lists of breeds predisposed to classic examples of canine autoimmune diseases, such as SLE (Systemic lupus erythematosus)^21,22^. A breed-specific form of immune-mediated polyarthritis has been described in Bernese mountain dogs^23^. Testing the canine anti-mouse antibodies for reactivity to autologous IgG would be one of the first steps towards a better understanding of their origin.

Solter and co-workers previously characterized heterophilic antibodies in canine plasma^10^. Their experimental setup differs from ours in that pooled canine plasma from unspecified subjects was used for their experiments. We have instead focused on characterizing anti-mouse antibodies in individual samples. This allows observation of differences in the characteristics of anti-mouse antibodies among individuals. Anti-mouse antibodies are often described as heterogeneous in nature^24^, which is reflected by our results. Multispecies reactivity was found in two of the samples whereas ten samples reacted only with mouse. The isotyping revealed that all of the major immunoglobulin classes normally present in canine serum can bind to mouse IgG. Our results also show that IgA, IgG and IgM with reactivity to mouse IgG can be found conjointly in one sample. There were also striking differences between individuals in the properties of anti-mouse antibodies. Four dogs were Fc-reactive and five were F(ab’)2-reactive None of the samples reacted both with Fc- and F(ab’)_2_-fragments. The selective binding to different fragments suggests that they might not originate from immunization through exposure, as this is expected to result in reactivity to both Fc- and F(ab’)_2_-fragments. This is supported by the results of the reference sample, which originates from immunization of chickens with whole mouse IgG, and which reacted with whole mouse IgG as well as with F(ab’)_2_-fragments and Fc-fragments. It is worth noting that all six Fc-reactive samples were collected from dogs with a diagnosis or clinical signs of disease. Only one of the five F(ab’)_2_-reactive sera was from a dog with diagnosis or clinical signs; a lipoma, generally not causing systemic disease. It has been speculated that F(ab’)_2_-reactivity precedes Fc-reactivity in certain human autoimmune diseases^25^ through spreading of the immune response along the immunoglobulin molecule. Intramolecular epitope spreading has been demonstrated for a number of human autoimmune diseases, including rheumatoid arthritis^26,27^. Prospective studies might provide insight on whether such epitope spreading occurs in dogs, and if F(ab’)_2_-reactive antibodies have potential to serve as prognostic markers for canine autoimmune disease.

The fragment-specific reactivity of anti-mouse antibodies characterizes some of their binding properties, but does not fully distinguish between anti-isotypic and anti-idiotypic antibodies. Anti-isotypic antibodies target the constant part of the antibody and are capable of binding to all molecules of an immunoglobulin class. This implies potential for interference in multiple immunoassays, because anti-isotypic antibodies are independent of the analyte detected by the assay. Moreover, there is significant structural homology between mammals in this region of IgG^28^, which means that anti-isotypic antibodies might react with IgG from several species commonly used to raise immunoassay antibodies. Anti-idiotypic antibodies bind to the idiotype within the variable part of another antibody and are generally not expected to cause multiple immunoassay interference. Fc-reactive antibodies are always anti-isotypic, because the Fc region is made up entirely of constant heavy chain domains. However, F(ab’)_2_-reactive antibodies can be either anti-isotypic or anti-idiotypic, since the Fab region of IgG consists of two constant and two variable domains. Because immunometric assays require simultaneous binding to two antibodies, the antibodies detected here are capable of binding both to non-immune polyclonal mouse IgG on the solid phase, and to a monoclonal mouse IgG1κ detection antibody that has undergone somatic hypermutation and has rearranged variable domains. Variable domains mainly contain amino acid sequences unique to antibodies with a certain antigen specificity. The murine B cell population consists of approximately 10^8 cells with a heterogeneous repertoire of immunoglobulin specificities^29,30^. This strongly suggests that the reactivity in the F(ab’)_2_-reactive antibodies is anti-isotypic. If this is true, the lack of binding to Fc-fragments indicates that the F(ab’)_2_-reactive antibodies target a relatively demarcated set of epitopes within the constant heavy or light chain domains of the Fab region, alternatively on or near the IgG hinge region. Naturally occurring anti-hinge antibodies in people have been described on numerous occassions^31–33^. The murine IgG hinge, present on all subclasses of IgG, is 13–22 amino acids in length^34^, which is sufficient to accommodate the paratope of any immunoglobulin molecule. One study concluded that human autoantibodies reacting with whole IgG and F(ab’)_2_-fragments, but not with Fc-fragments, recognize determinants on or near the hinge region of IgG, on the IgG Fdγ region or on the IgGλ light chains^35^. All of these options except IgGλ light chains are possible in our case, since we used a detection antibody with IgGκ light chains. The signal may also be supplemented by binding to a nonspecific immunoglobulin fraction of the detection antibody, which is always retained in antiserum purified with protein G. Anti-idiotypic reactivity remains a possibility, since there are cross-reactive idiotypes (sequences shared by different individuals) in inbred mouse strains^36,37^. The idiotype of an antibody also contains a framework region with less variability than the complementarity determining region. It has previously been suggested that F(ab’)_2_-reactive antibodies in people recognize a repertoire of idiotypes commonly found in pooled IgG^38^. Counterintuitively, three samples reacted with whole IgG, but with neither Fc nor F(ab’)_2_-fragments. This may suggest binding to epitopes on or near the hinge region of the Fc and Fab regions that are lost or altered by the cleaving of fragments with pepsin. Pepsin cleaves IgG within the lower part of the hinge region^39^. Furthermore, attachment to plastic surfaces frequently causes conformational changes to capture antibodies^40^, which might expose or conceal antigenic regions depending on the physical orientation of the antibody and its interaction with polystyrene.

Removing the Fc region from immunoassay antibodies would omit all but two of the constant IgG domains, and is sometimes suggested to be an efficient strategy to reduce immunoassay interference^14^. Other researchers have found F(ab’)_2_-reactive antibodies to be rare, implying that the use of F(ab’)_2_-fragments would eliminate the vast majority of all antibody interference^15,41^. By contrast, our findings, as well as those of others^42,43^ suggest that the heterogeneity of interfering antibodies makes the efficiency of this strategy questionable. According to our results, use of F(ab’)_2_-fragments would only mitigate interference in roughly half of dogs with anti-mouse antibodies. Engineering of single-chain variable fragment (scFv) antibodies circumvents anti-isotypic binding^41^, but would entail significant additional production costs. An alternative strategy to avoid interference from canine anti-mouse antibodies may be to combine immunoassay antibodies from two species, since only two of 14 samples exhibited multispecies reactivity. However, monoclonal antibodies are only widely available from mouse, and to a lesser extent rat and rabbit, which limits the combinations and strategies that can be used to set up an assay with monoclonal antibodies from two species. In the cross-reactivity experiment, polyclonal IgG on the solid phase was combined with a monoclonal detection antibody raised in mouse. This setup can be used to combine the sensitivity of a polyclonal antibody with the specificity of a monoclonal antibody. Increasing the phylogenetic distance between the two species used to raise the antibodies will narrow the spectrum of potential cross-linking antibodies^44–46^. For example, endogenous antibodies are unlikely to be capable of binding immunoglobulins of mammalian and avian origin simultaneously. In our experiment, a combination of polyclonal chicken IgY on the solid phase with a monoclonal mouse antibody for detection resulted in the least amount of nonspecific reactivity overall. If the interfering antibodies should be able to bridge immunoglobulins from two different species, it can be difficult to gauge which blocking agent to use. Preferably non-immune IgG from both species is combined, but if this is not available, IgG resembling either of the capture and detection IgGs may be sufficient to eliminate the interference. We noted that non-immune goat IgG, mouse IgG and a mixture of goat and mouse IgG all dramatically reduced the signal in the sample that reacted strongest with goat IgG on the solid phase and with conjugated mouse IgG free in solution. However, the results may vary depending on antibody concentrations and conditions within each specific assay, as well as the concentrations of the blocking antibody.

If monoclonal antibodies are critical to the assay design, combining two different subclasses of mouse IgG is another potential strategy for counteracting immunoassay interference in dogs, since there is documentation of human anti-mouse antibodies with specificity for different mouse IgG subclasses^47^. We did not attempt to investigate subclass specificity in this study, but it is within the realm of possibility that canine anti-mouse antibodies share this attribute with equivalent antibodies in people. As covered elsewhere in the discussion, the IgG hinge is one of the candidate binding sites for canine anti-mouse antibodies. Much of the structural variability between IgG from different species, notably between dogs and mice, is located in the hinge region^34^. This makes the murine IgG hinge immunogenic to dogs, and because much of the variability between subclasses of IgG is also located in the hinge region, anti-hinge antibodies are often subclass-specific^48,49^. However, not all anti-mouse antibodies in people are subclass-specific, and there are amino acid sequences expected to be immunogenic to dogs distributed all over the murine IgG molecule, including on subclass-shared segments, so it may be a strategy with limited potential on its own.

The isotyping experiment is contingent on the endogenous antibodies not binding to goat IgG, as nonspecific binding to the anti-canine detection antibodies (all of them raised in goat) would drown the specific signal in background and make it impossible to distinguish between the antibody isotypes. The two cross-reactive samples did not pass this criterion and were therefore excluded from this experiment. One of the isotyped sera was shown to contain Fc-reactive IgM antibodies. This is consistent with the traditional definition of a rheumatoid factor. The definition of rheumatoid factors is nowadays often broadened to include other antibody classes, which means that the other detected Fc-reactive antibodies also might be rheumatoid factors. Presence of IgM could also indicate recent exposure to antigen, since IgM is the main isotype secreted during the primary immune response. The causative agent is difficult to identify, because of the sheer number of everyday events that can trigger antibody formation. Vaccinations, pet keeping and food ingestion are some examples of suggested triggers for heterophilic antibodies in people^50^. Following class switching, the IgM response is supplemented by an increase in IgG levels, and presence of interfering IgG antibodies may reflect a secondary immune response to antigen that overrides the IgM production. Compared to IgG and IgM, the role of serum IgA is obscure, but involves immunoregulatory functions^51^. IgM generally binds antigen with lower affinity than IgA and IgG, but may use its multimeric structure to gain a higher combined binding strength (avidity). The IgG molecule is monomeric with two functional antigen-binding sites and in dogs, serum IgA is dimeric with four binding sites^52^. The IgM molecule is a pentamer with ten binding sites. Consistent with most mammals, IgG is the most abundant immunoglobulin in canine serum (average levels 1000–2000 mg/dL), followed by IgM (70–270 mg/dL) and IgA (20–150 mg/dL)^53–55^. Heterophilic IgA, IgG and IgM antibodies have all been demonstrated previously^4^ and rheumatoid factors of all these immunoglobulin classes are known to exist in people^56,57^. To date, only IgA and IgM rheumatoid factors have been described in dogs^58,59^, but this may be related to limitations in the methods used to study them^60^. It was found by Solter and co-workers that the majority of heterophilic antibodies in pooled canine plasma was IgG^10^.

Based on the accumulated findings of the study, there are considerable similarities between canine anti-mouse antibodies and equivalent antibodies in people. Both are heterogeneous in their composition and nature and there are individual differences in their properties. However, F(ab’)_2_-reactive antibodies may be more frequent in dogs than in people. The dog breed difference in prevalence of anti-mouse antibodies is of interest from an etiological point of view and could have tangible effects on patient safety if proper care is not undertaken to prevent immunoassay interference. To minimize the risk of antibody interference, manufacturers and researchers setting up immunoassays have to take several factors into account. A practical suggestion based on the findings in this study, and on previous knowledge, would be to use capture and detection IgG from two species that are distantly removed phylogenetically, mix non-immune IgG from both species, heat-treat the mixture^14^ and add it to the sample incubation buffer. These basic measures are cheaper, less cumbersome, and for canine samples quite possibly more effective against interference in immunometric assays than preparation of F(ab’)_2_-fragments. However, the heterogeneous nature of canine anti-mouse antibodies and the variety of immunoassays used in research and patient care makes it unlikely that any laboratory-based counteractions will be universally effective in eliminating antibody interference. It is therefore recommended that anti-mouse antibodies are considered as a potential source of dubious test results in immunoassay analysis of canine samples. In a clinical setting, it is important that clinicians are attentive to test results deviating from the clinical picture, and that any suspicion of erroneous test results is communicated to the analyzing laboratory.

## Conclusions

Anti-mouse antibodies in dogs consist of all three major immunoglobulin classes present in dog serum, target different fragments of the murine IgG molecule, and are capable of cross-reacting with IgG from a variety of species. Their properties vary among individuals, and there are breed differences in their prevalence. Due to the heterogeneity of canine anti-mouse antibodies, a combination of countermeasures is likely required to mitigate antibody interference in dogs.

## Methods

### Canine samples

Samples were obtained from two main sources: 1) The routine laboratory analysis at the University Animal Hospital in Uppsala or Djursjukhuset in Jönköping, Sweden and 2) Dogs belonging to volunteering private owners, recruited through online advertising. These samples were collected at local clinics in various parts of the country and sent to our laboratory in Uppsala. All samples were frozen at −20 °C before analysis. Exclusion criteria were clearly visible signs of hemolysis, bilirubinemia, or lipemia. Ethical permission was obtained from the Uppsala Animal Ethical Committee (C136/13) and all experiments were performed in accordance with relevant guidelines and regulations.

### Screening for breed differences

Flat-bottom 96-well polystyrene microtiter plates were incubated overnight at 4 °C with purified non-immunized mouse IgG as whole molecules, Fc-fragments and F(ab’)_2_-fragments (Jackson ImmunoResearch, Ely, UK), respectively (all 2 µg/mL), all diluted in NaHCO_3_ (0.05 M, pH 9.6). The plates were washed 4 times with Tween-20 diluted in PBS (200 µL, 0.05%). Serum samples were incubated as singletons and the reference sample in duplicate for 90 minutes at RT. The samples were assayed undiluted. Chicken anti-mouse IgG (Immunsystem AB, Uppsala, Sweden) was used as reference sample and positive control at a dilution of 1:1,600. Two wells on each plate were incubated with PBS as negative control.

After another cycle of 4 washes, the plates were incubated with monoclonal HRP-conjugated mouse anti-human CEA IgG diluted 1:200 (MBS592181, Mybiosource, San Diego, CA, USA) for 90 minutes, washed again, and finally incubated with TMB for approximately 8 minutes in the dark before the reaction was stopped with H_2_SO_4_ (100 µL). Plates were read at 450 nanometers in a photometric microplate reader. All incubations were made with volumes of 50 µL except for the TMB, of which 100 µL was added. The cut-off for distinguishing a positive from a negative result was set to the assay LoD (based on the mean of 20 replicates of the 0-calibrator) + 4 SD for all assays used in this study. The signal-to-noise (S/N) ratio for each assay was evaluated by dividing the median of the positive sample OD values with the median of the negative control values.

A sample positive in at least one of the Whole IgG, Fc and F(ab’)_2_ assays was considered positive for anti-mouse antibodies.

### Cross-reactivity to non-murine IgG and blocking of cross-reactive antibodies

The cross-reactivity experiment was performed as above, with the mouse capture antibody substituted for goat, sheep (both from Jackson ImmunoResearch), rabbit IgG (Sigma Aldrich, St. Louis, MO, USA) and chicken IgY (Immunsystem). All mammalian antibodies were coated in NaHCO_3_ as described above. For the chicken IgY, PBS (0.01 M, pH 7.4) was used as coating buffer. Blocking was performed by incubating the sample with different concentrations of the same non-immune goat IgG and mouse IgG described previously for 30 minutes before assaying the blocked sample. The native sample was diluted 1:2 with PBS to compensate for any dilution effects due to addition of IgG. Chicken anti-mouse IgG diluted 1:400 was used as positive control.

### Antibody isotyping

For isotyping the antibodies, microtiter plates were coated with mouse IgG and washed as previously described, but the detection was performed with biotinylated Fc-specific goat anti-canine IgA, IgM (both from Nordic-MUbio, Susteren, The Netherlands), and IgG (Sigma Aldrich), all diluted 1:10,000. The wells were incubated with 1:500 streptavidin-HRP for 90 minutes. The plates were coated, washed and read as previously described.

### Statistical calculations

Differences in breed prevalence were tested with a two-sample test for equality of proportions with continuity correction, two-tailed, α = 0.05. Statistical analysis was performed using R software, version 3.3.3 (R Core Team, Vienna, Austria).

## Data Availability

All data generated or analyzed during this study are included in the submitted manuscript.
